# Fixation and Visualization of Full Protein Corona on Lipid Surface of Composite Nanoconstruction

**DOI:** 10.3390/nano13243094

**Published:** 2023-12-07

**Authors:** Anna V. Epanchintseva, Julia E. Poletaeva, Irina A. Bakhno, Vladimir V. Belov, Alina E. Grigor’eva, Svetlana V. Baranova, Elena I. Ryabchikova, Ilya S. Dovydenko

**Affiliations:** Institute of Chemical Biology and Fundamental Medicine SB RAS, 630090 Novosibirsk, Russia; annaepanch@niboch.nsc.ru (A.V.E.); fabaceae@yandex.ru (J.E.P.); uta-15@mail.ru (I.A.B.); v.belov@g.nsu.ru (V.V.B.); feabelit@mail.ru (A.E.G.); swb@niboch.nsc.ru (S.V.B.)

**Keywords:** protein corona, multilevel nanoconstructions, serum proteins, photomodification, fixation of full corona, full corona visualization

## Abstract

Spontaneous sorption of proteins on the nanoparticles’ surface leads to the fact that nanoparticles in biological media are always enveloped by a layer of proteins—the protein corona. Corona proteins affect the properties of nanoparticles and their behavior in a biological environment. In this regard, knowledge about the composition of the corona is a necessary element for the development of nanomedicine. Because proteins have different sorption efficacy, isolating particles with a full corona and characterizing the full corona is challenging. In this study, we propose a photo-activated cross-linker for full protein corona fixation. We believe that the application of our proposed approach will make it possible to capture and visualize the full corona on nanoparticles coated with a lipid shell.

## 1. Introduction

The discovery of the phenomenon of a protein corona covering any nanoparticle (NP) in any biological fluid has undoubtedly complicated the development of therapeutic nanodrugs. On the other hand, developers may get the opportunity to “control” the delivery and action of nanodrugs by modulating the composition of the protein corona. 

Hundreds of papers have been published on the study of the protein corona on various NPs in a variety of biological fluids, but the ability to “control” nanodrugs through changes in the protein corona remains potential. The main reason for this is the layered structure of the protein corona: proteins in the first layer are irreversibly adsorbed onto the NP surface (hard corona, HC), while proteins in the second layer (soft corona, SC) dynamically bind to the proteins on the HC and NPs surfaces. The composition of the SC easily changes with the slightest influence, even when stirring the NP suspension [1,2]. The interest of researchers in the SC is due to data on its decisive role in determining the nature of the interaction of NPs with cells. It is the components of the SC, and not the chemical composition of the NP surface, that determine the cell response to NP exposure and, accordingly, the launch of certain processes in the body [3,4]. The need to know the composition of the SC to analyze the behavior of NPs in different biological environments is becoming more and more urgent as information about its importance in realizing the effects of nanomedicines accumulates [4,5,6]. The variability of the SC under the influence of external factors complicates the task of determining its composition; a few methods have been proposed.

Determining the composition of the SC involves two independent tasks: (1) fixation of the SC, which is extremely labile, and (2) its isolation and analysis. Several approaches have been proposed for the isolation and analysis of SC proteins. Automated analysis of full corona proteins on magnetic NPs without the use of ultracentrifugation [7]. Affinity chromatography uses an anti-PEG single-chain variable fragment to determine the protein composition of PEG-modified NPs [8]. Asymmetric flow field-flow fractionation (AF4) has also been used to preserve the SC [9]. Another way to fix SC proteins is by cross-linking proteins using click chemistry [10]. The essence of this method is to alternately modify NPs bearing a HC with an azide-containing linker and serum proteins with a DABCO-bearing linker. By mixing the modified NP/corona complex and serum, the authors were able to covalently immobilize SC proteins onto the surface of the NP. 

This elegant approach makes it possible, using simple washes, to easily remove unbound proteins from the biological environment without losing the components of the SC. However, despite all the advantages of this approach, we believe that the chosen fixation method is of limited use. Thus, fixation is possible only when two modifiers are spatially close together, so the presence of steric hindrances can lead to data loss when analyzing the corona. Also, proteins with low adsorption capacity on the NP surface will remain “unaccounted for”.

In this work, we tried another approach by replacing click chemistry with a photoactivatable cross-linker (PACL). PACL is a molecule bearing two orthogonal reactive groups: (i) a maleimide residue to modify serum proteins at thiol groups; (ii) an arylnitroazide group to form covalent cross-links between proteins and nanoparticles under UV irradiation. In this case, only serum proteins are modified, which are then fixed on the surface of model nanocomposite particles due to short-term UV irradiation.

As an experimental model, we used a system of 10% fetal bovine serum-multi-level nanoconstructs (MLNCs). MLNCs are composite nanoparticles designed to deliver siRNA into cells. The assembly process of MLNCs and their physicochemical properties have been studied in detail previously [11,12]. To precondense siRNA, we use gold nanoparticles (AuNPs), which can reversibly adsorb siRNA to their surface. The noncovalent complex of siRNA with AuNP serves as the core of the MLNC. One MLNC can contain from one to several cores, which remain individual. The core or group of cores is enveloped by a lipid membrane, which protects siRNA from nucleases as well as from premature desorption from the AuNP surface.

Self-assembly of MLNC occurs due to electrostatic interaction between the cores bearing the negative charge of siRNA and the lipid film with the positively charged pH-sensitive lipidoid 2-[[4-Dodecylamino-6-oleylamino-1,3,5-triazine-2yl]-(2-hydroxyethyl)amino]ethanol. 

When MLNC enters a biological fluid, only the lipid envelope will interact with the components of the fluid and, thereby, determine the composition of the corona.

In this work, MLNC is a model system to study the formation of a corona on the surface of lipid particles. Despite its apparent complexity, the construct has a number of advantages over the use of simple lipid vesicles. MLNCs have a heavy core containing AuNPs, so they can be easily purified from medium components by low-speed centrifugation in viscous solutions. The presence of AuNPs makes MLNC suspensions colored, which allows the researcher to accurately collect the target fraction of nanoparticles. The composition of the lipid membrane can also be varied; different cationic and pH-sensitive lipids can be used in this system, which in the future will make it possible to determine the corona for different lipid compositions of MLNCs.

## 2. Materials and Methods

### 2.1. Chemicals

Disodium phosphate dihydrate (NaH_2_PO_4_·2H_2_O) and monosodium phosphate dodecahydrate (Na_2_HPO_4_·12H_2_O) were purchased from Reatex (Moscow, Russia). Sucrose, DMSO, and Coomassie R-250 Brilliant Blue were bought from Panreac (Barcelona, Spain). Glycerol and acrylamide were obtained from Applichem (Darmstadt, Germany), and bis-acrylamide was obtained from Amresco (Solon, OH, USA). Tris hydrochloride, SDS, and bromophenol blue (BP) were purchased from Helicon (Moscow, Russia). Glycine and bovine serum albumin (BSA) were purchased from Sigma-Aldrich Chemie Gmbh (Munich, Germany). Precision Plus Protein Kaleidoscope: Prestained Protein Standards Bio-Rad (Hercules, CA, USA) was used as a marker of the molecular weight of proteins. Fetal bovine serum (FBS) A3160801 from Thermo Fisher Scientific (Waltham, MA, USA) was used for all serum experiments. Experimental water was purified using a Simplicity 185 water purification system from Millipore (Burlington, MA, USA) and had a resistivity of 18.2 MΩ·cm at 25 °C. PACL (4-azido-N-[3-[3-(2,5-dioxopyrrol-1-yl)propanoylamino]propyl]-2-nitro-benzamide) was synthesized in the laboratory of organic synthesis of ICBFM SB RAS (Novosibirsk, Russia). Cy-5-labeled albumin was kindly provided by Dr. Alexey S. Chubarov from the laboratory of structured biology at ICBFM SB RAS (Novosibirsk, Russia).

### 2.2. Assembly of the MLNC

The assembly of MLNCs consists of three steps: synthesis of gold nanoparticles (AuNPs); core preparation (non-covalent coating of AuNPs with siRNA molecules); and enveloping the core in a lipid shell doped with a cell-penetrating peptide. The AuNPs were synthesized as described in [13]. The preparation and characterization of cores from AuNP and siRNA are described in detail in [11,12]. The procedure for MLNC assembly is described in [11]. The resulting preparation will be further referred to as the “initial” MLNC. 

### 2.3. Modification of FBS or BSA Proteins with PACL

Approximately 50% FBS or BSA solution (21 mg/mL) in 1 mM phosphate buffer (PB) was mixed with 12 µL of 0.5 M PACL in DMSO and incubated at 20 °C with constant stirring (400 rpm) for two hours. Excess PACL was removed using Bio-Spin 6 columns, Bio-Rad (Hercules, CA, USA), according to the manufacturer’s protocol. Since PACL is sensitive to light, all work with this compound was carried out in a dark room illuminated by a red lamp.

### 2.4. Incubation of MLNCs with Solutions of FBS or BSA

One volume of MLNC (2.5 nM, determined by AuNPs optical density) was added to a quarter volume of a 50% solution of FBS or BSA (21 mg/mL) in 1 mM PB. The mixture was kept for 15 min at 25 °C. When PACL-modified FBS or Cy-5-labeled albumin were used, incubation was performed in the dark. 

To bind PACL-modified FBS proteins to the surface of MLNCs, a mixture of MLNCs with PACL-modified FBS was exposed to UV light with a wavelength of 310 nm and 8.2 mW/cm^2^ for 1 min. 

### 2.5. Isolation of MLNCs

MLNCs incubated with FBS with/without UV irradiation (Section 2.4) were cleared of unbound proteins by centrifugation in 75% glycerol or 58% sucrose containing 1 mM PB at 2000× *g* and 25 °C for 15 min as described in [12]. 

Colored fractions containing the resulting modified MLNCs were collected and subjected to subsequent purification, concentration, and analysis. A similar treatment was carried out in the case of using glycerol or sucrose solutions containing 10% serum as a viscous medium. The prepared preparations will be designated as “isolated” MLNCs. 

### 2.6. Washing and Concentration of Isolated MNLCs

To wash MLNC from unbounded proteins and increase the concentration of corona-bearing MLNC, identical MLNC suspensions collected after isolation were transferred to a 15 mL tube. The suspension was diluted 3.75 times by 1 mM PB and centrifuged at 3000× *g* and 25 °C for 10 min, and the supernatant was removed. The pellet of MLNCs was washed with 1.5 mL of 1 mM PB and centrifuged again at 3000× *g* and 25 °C for 10 min. The supernatant was removed, and the procedure was repeated two more times. After four washes, the MLNC pellet was ready for analysis.

### 2.7. Dynamic Light Scattering

MLNC suspensions before and after contact with serum were analyzed using a Malvern Zetasizer Nano, Malvern Instruments (Worcestershire, UK), according to the manufacturer’s instructions to determine the ζ-potential and hydrodynamic diameter of the particles. Each measurement was carried out in thirty runs. 

### 2.8. Optical Extinction Spectra

To verify the colloidal stability of MLNCs, we performed UV–Vis spectroscopy. Optical adsorption spectra of the MLNCs and AuNPs were recorded on a Clariostar plate fluorimeter, BMG Labtech (Ortenberg, Germany), in the range 400–800 nm according to the manufacturer’s instructions. Preservation of the colloidal stability of the samples during the procedure was confirmed by the absence of a peak in the range 600–700 nm.

### 2.9. Fluorescence Spectroscopy

Measurements of Cy5-fluorescence were carried out in 250 mL aliquots using a Clariostar plate fluorimeter (BMG Labtech, Germany) upon excitation at 610 nm and emission detection at 675 nm. 

### 2.10. Electrophoresis 

MLNCs before and after interaction with serum were characterized by electrophoretic mobility in agarose gel. To perform this, samples containing 5 µL of MLNCs (0.5 pmol) and 0.5 µL of glycerol/deionized water (1:1, *v*/*v*) were loaded into the wells of a 0.8% agarose in Tris-glycine buffer (250 mM Glycine, 25 mM Tris, Ph 8.3). Electrophoresis was carried out for 30 min at 5 V/cm. Images were obtained using an Epson Perfection 4990 Photo (Epson, Suva, Japan) scanner.

Proteins bounded to the MLNC surface were analyzed by sodium dodecyl sulfate-polyacrylamide gel electrophoresis using 7% PAAG under Laemmli conditions with subsequent Coomassie Brilliant Blue staining.

### 2.11. MALDI Mass Spectrometry

The mass spectra were recorded on an Autoflex Speed mass spectrometer (Bruker Daltonics, Bremen, Germany) in the Core Facility of Mass Spectrometric Analysis (ICBFM SB RAS, Novosibirsk, Russia). The 2,5-DHAP (2,5-Dihydroxy actetophenone) matrix (Sigma-Aldrich, St. Louis, MO, USA) was prepared by the dried-droplet method. The equal volumes of sample, 2% TFA (Trifluoroacetic acid) (Sigma-Aldrich, St. Louis, MO, USA), and matrix solution were pre-mixed, and one microliter of the mixture was manually spotted on an MTP 384 ground steel target (Bruker Daltonics, Bremen, Germany) and dried at ambient temperature. MS spectra were recorded in the positive reflective mode in the range 5–150 kDa by means of the FlexControl Version 3.3 software (Bruker Daltonics, Bremen, Germany, status on 1 November 2023) and were analyzed in the FlexAnalysis Version 3.3 and Mmass software Version 5.5.0 (mmass.org, status on 1 November 2023). The prepared MTP standard target (Bruker Daltonics, Bremen, Germany) was used for external calibration. 

### 2.12. Transmission Electron Microscopy

All reagents for TEM studies were purchased from EMS (Houston, TX, USA). A drop of any sample was applied for 1 min on a copper 200-mesh grid covered by formvar film stabilized with carbon. The grid was then placed on a drop of 0.5% aqueous uranyl acetate solution for 5–10 s. At each stage, excess liquid was removed with filter paper. Grids were examined using a Jem1400 transmission electron microscope (Jeol, Tokyo, Japan), and images were collected using a Veleta digital camera (EM SIS, Münster, Germany).

## 3. Results

In this work, we used the MLNCs that we had previously constructed, for which the physicochemical characteristics and conditions for isolation from reaction mixtures after synthesis, purification, and concentration were determined, ensuring the production of high-purity preparations. The structure and physicochemical properties of MLNCs determined the choice of these particles as the carriers of the protein corona. In particular, the lipid envelope of MLNCs is preserved during low-speed centrifugation in viscous media (75% aqueous glycerol (GS) or 58% aqueous sucrose (SS)). The presence of AuNPs in the MLNC composition provides red color to the suspensions, which makes it easy to collect the MLNC fraction from a transparent viscous medium [11].

### 3.1. Confirmation of the Formation of a Protein Corona on the Surface of MLNCs

**Figure 1:**
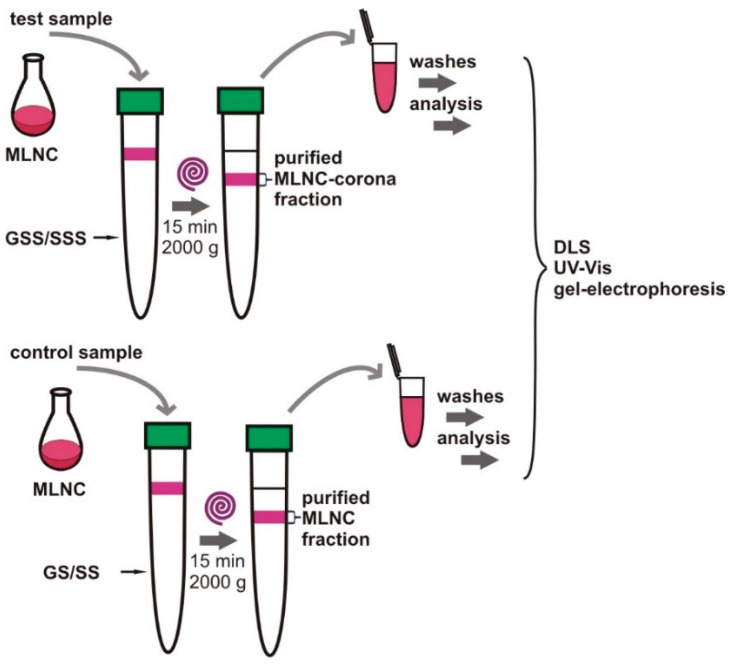
Scheme of experiments to obtain MLNCs bearing the protein corona. Top: To obtain the corona, the MLNC suspension was applied to the surface of glycerol supplemented with serum solution (GSS) or sucrose solution supplemented with serum (SSS) and centrifuged. The colored MLNC/PC fraction was collected, washed, and analyzed. Bottom: Control of corona formation. The MLNC suspension was processed identically to the top one, but without serum. The samples were analyzed in parallel and identically.

To obtain the protein corona, we used FBS, the proteins of which should form a protein corona on the surface of the particles [14]. The presence of the corona should lead to a change in the physicochemical characteristics of the MLNCs, and we examined these changes to confirm the formation of a corona by FBS proteins on the surface of MUNCs. Figure 1 shows experiments that resulted in the formation of a protein corona on the surface of MLNCs.

At the first stage, we used GS and SS solutions containing 10% FBS, hereinafter referred to as GSS and SSS. A total of 1 mL of MLNC suspension, prepared as described previously [11], was applied to the surface of GSS or SSS solutions. After 15 min of centrifugation at 2000× *g*, the colored fraction of MLNCs presumably bearing the protein corona (MLNC/PCs) was collected. MLNCs without corona isolated after centrifugation in a viscous medium without serum were used as a control.

The hydrodynamic diameter of the MLNC/PCs increased to 234.8 ± 62.4 nm (GSS) and 219.5 ± 58.3 nm (SSS) compared to the control sample (156.2 ± 47 nm).

**Figure 2:**
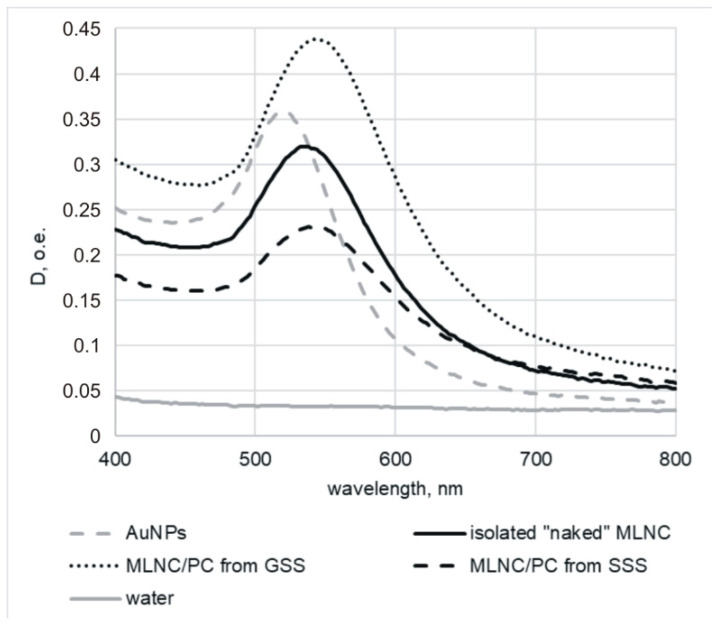
Absorption spectra of MLNCs isolated from viscous solutions with serum. The wavelengths of absorbed light are plotted along the abscissa axis, and the optical density is plotted along the ordinate axis.

Optical absorption spectroscopy showed an 8 nm shift in absorption maxima for MLNC/PCs obtained by centrifugation with GSS or SSS compared to the control (Figure 2). The absorption maximum for particles that were in contact with serum was 544 nm, regardless of the type of viscous medium, while for control MLNCs it was 536 nm. This shift is typical for changes in the surface characteristics of MLNCs. We previously observed a similar shift when the core was “wrapped” in a lipid envelope [11]. 

**Figure 3:**
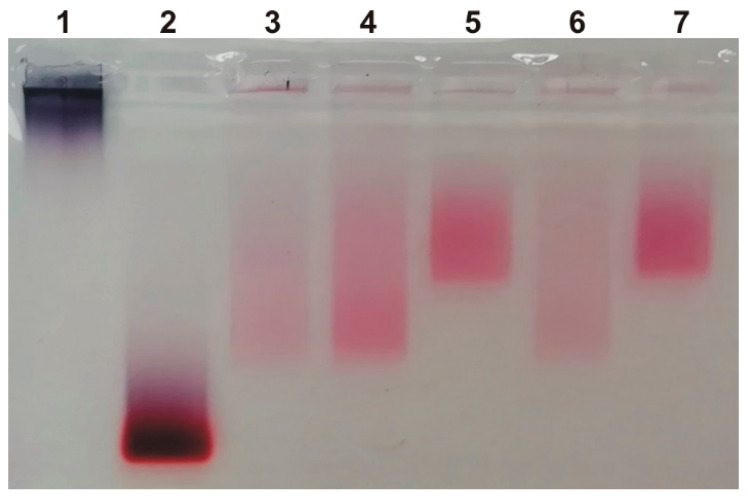
Agarose gel electrophoregram of MLNCs before and after contact with serum proteins: 1—AuNPs; 2—cores of MLNCs (AuNPs covered with siRNA); 3—initial MLNCs; 4—isolated MLNCs from GS; 5—MLNC/PC from GSS; 6—MLNCs from SS; 7—MLNC/PC from SSS.

The differences between the initial and isolated MLNCs and MLNC/PCs were also confirmed by gel electrophoresis (Figure 3). Initial MLNCs have exactly the same mobility profile as isolated MLNCs (lanes 3 and 4). Centrifugation in a viscous medium eliminates “empty” lipid particles; they are colorless and not visible in the electropherogram in the lane with the initial MLNCs (lane 3). MLNC/PCs obtained by centrifugation in GSS (lane 5) or SSS (lane 7) have lower mobility, apparently due to the binding of MLNC to serum proteins.

The obtained data on changes in absorption spectra, hydrodynamic diameter, and electrophoretic mobility demonstrate changes in MLNCs, confirming the formation of a protein corona on their surface. This corona, in accordance with existing ideas, is a “full” corona, consisting of a HC and SC. The protein corona was formed in both GSS and SSS media. The use of one or another type of viscous medium does not affect the physicochemical properties of the MLNCs, so we further used a 75% GSS to provide a higher yield.

### 3.2. Isolation of MLNCs Bearing a Hard Protein Corona

**Figure 4:**
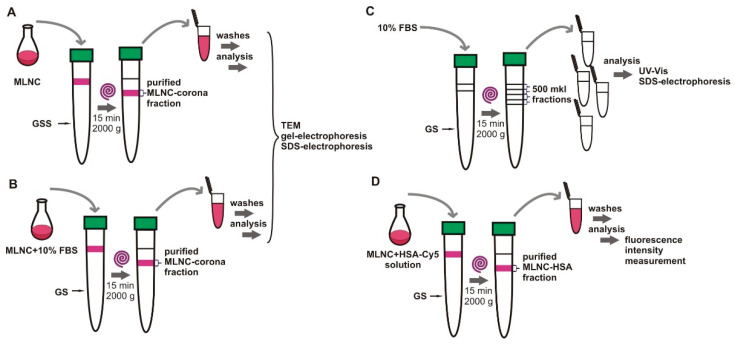
Scheme of experiments aimed at obtaining MLNC/HC preparations purified from unbound serum proteins. Removing free serum proteins from viscous medium using centrifugation: (**A**) (control, MLNCs without serum); (**B**) (MLNCs preincubated in 10% serum). The obtained colored fraction was washed and analyzed. (**C**)—Testing the ability of serum proteins to diffuse into a viscous solution: a 10% FBS solution was applied to the surface of the GS, and after centrifugation, 0.5 mL fractions were collected and analyzed. (**D**)—determination of the number of washings using Cy5-labeled albumin solution. The samples were analyzed in parallel and identically.

Using GSS or SSS, we showed that MLNCs acquire a protein corona when incubated with serum, and the next step toward fixing the protein corona was to isolate MLNCs bearing a hard corona. The HC is composed of proteins with high binding affinity to the nanoparticle surface, while the SC proteins are labile and will inevitably be lost during the purification process [15,16]. This circumstance made it possible to reduce the task of obtaining purified MLNC/HCs to removing proteins not associated with particles from the reaction mixture. Figure 4 shows a scheme of experiments aimed at obtaining MLNC/HC preparations purified from unbound serum proteins.

#### 3.2.1. Selection of Conditions for Purification of MLNC/HCs from Unbound Serum Proteins

**Figure 5:**
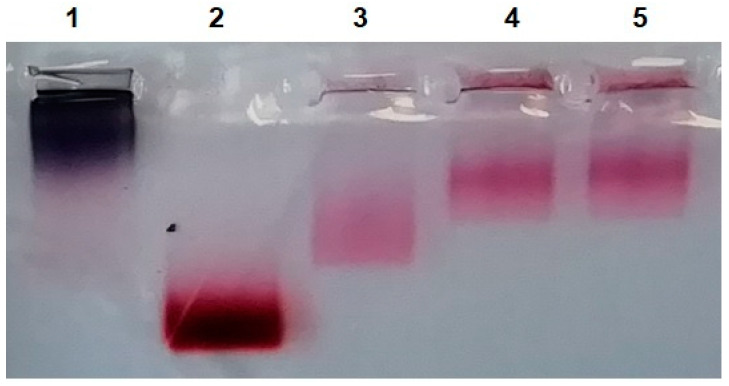
Agarose gel electrophoregram of isolated MLNCs. 1—AuNPs; 2—cores of MLNCs (AuNPs covered with siRNA); 3—MLNCs isolated from GS; 4—MLNC/HC obtained by pre-incubation of MLNC in FBS and subsequent isolation using GS; 5—MLNC/PC obtained using GSS.

We understood that if MLNC/HC is isolated from viscous media, the preparation will be contaminated with unbound proteins; therefore, to isolate MLNC/HC, we removed FBS from the viscous medium. However, it was not clear whether HC would be retained on the surface of MLNCs. To check the preservation of the HC, the particles were incubated in 10% serum before isolation, and then this mixture was applied to the surface of the GS and centrifuged (Figure 4B). The applicability of this approach was confirmed by gel electrophoresis. Two samples were prepared: (1) MLNC/PCs obtained using GSS, and (2) MLNC/HCs obtained by pre-incubation of MLNCs in FBS and subsequent isolation using GS. In the electrophoregram (Figure 5), we see that the mobility of both preparations is the same and noticeably lower than for MLNCs isolated from GS. 

**Figure 6:**
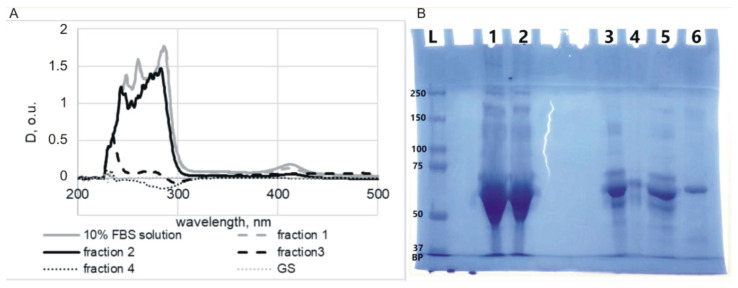
Analysis of serum proteins’ ability to diffuse in GS. (**A**) Absorption spectra of the collected fractions (0.5 mL each). (**B**) SDS-PAGE of selected fractions: L—marker of molecular weights of proteins; 1—fraction 1; 2—fraction 2; 3—fraction 3; 4—fraction 4; 5—FBS; 6—BSA. BP—bromophenol blue.

Thus, to obtain MLNC/HCs and remove excess components of biological fluid (serum proteins), MLNCs can be pre-incubated in the serum, and then HC-carrying particles can be isolated using GS. We also tested whether serum proteins could diffuse into the GS along with the MLNCs during centrifugation (Figure 4C). To perform this, a 10% FBS solution was applied to the surface of the GS, and after centrifugation, 0.5 mL fractions were collected and analyzed for the presence of proteins using UV-vis spectrometry and SDS PAGE (Figure 6). The fourth fraction corresponded to the sample in which MLNCs were concentrated.

The highest concentration of protein material was observed in the first two fractions. In fraction three, the protein concentration decreased significantly, and in fraction four, the protein was not detected by spectrometry (Figure 6A). However, the electrophoresis detected contamination with protein components of the serum, and the presence of protein in fraction four was clearly visible (Figure 6B). This suggests that the proteins diffuse into the GS during centrifugation. Therefore, additional steps were required to obtain a pure MLNC/HC preparation. 

We observed that changes indicative of corona formation on MLNCs in GSS persist after incubation with FBS and isolation from GS, as well as during electrophoretic analysis (Figure 3 and Figure 5). This indicates a very low rate of desorption of serum components bound to the MLNC surface. Therefore, without fear of loss of HC, you can resort to additional washing of the MLNA preparation to remove excess unbound serum proteins. 

To determine the wash conditions for MLNC/HCs (Figure 4D), an albumin solution (41.2 mg/mL) was used, which corresponds to the total protein concentration in the serum. To control the effectiveness of washing, for these experiments we used 0.8% Cy5-labeled albumin. The albumin solution was mixed with initial MLNCs 1 to 10, the mixture was uploaded on the surface of the GS, and the MLNC fraction was isolated according to the standard protocol. Then, MLNCs were washed sequentially with 1 mM PB; after each wash, the supernatants were collected, and the fluorescence intensity was determined (Table 1).

**Figure 7:**
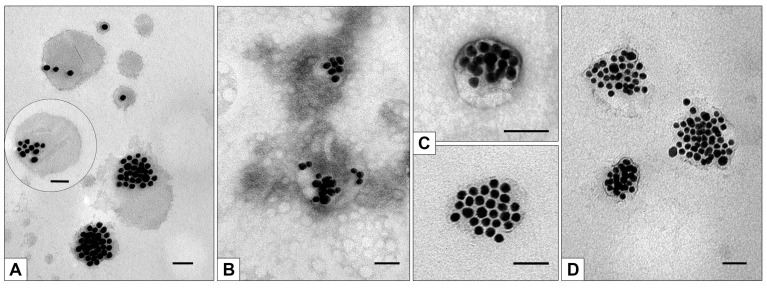
Representative images of: (**A**)—initial MLNCs (the insert shows a variant of MLNCs); (**B**)—MLNCs incubated with 10% FBS; (**C**,**D**)—MLNC/HC after 4 washings. “Grey” material on (**B**) represents unbound serum components. The length of the scale bars corresponds to 50 nm.

To control the state of MLNCs, preparations obtained after washing were studied in TEM. Figure 7 shows images of the initial MLNCs, MLNCs incubated with 10% FBS, and MLNCs obtained as a result of the above-described procedures for washing MLNC/HCs from unbound proteins. 

It can be seen that MLNCs maintained their integrity after four washings (Figure 7C,D), and unbound serum components around the MLNCs (Figure 7B) disappeared. Based on the data obtained by two methods, we may conclude that four washes of the MLNC pellet are sufficient to completely remove unbound protein. Thus, we have developed a method for obtaining fully purified MLNC/HCs for further research.

#### 3.2.2. Determining the Quantity of MLNCs Needed to Study Hard Corona

**Figure 8:**
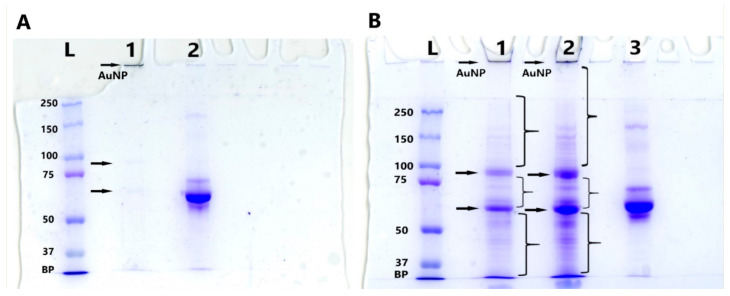
Scanned SDS-PAGE image. (**A**) L—marker of protein molecular weights; 1—two MLNC/HC preparations; 2—FBS. (**B**) L—marker of protein molecular weights; 1—four MLNC/HC preparations; 2—eight MLNC/HC preparations; 3—FBS. Arrows indicate major bands; curly brackets indicate minor bands; BP—bromophenol blue.

It is well known that each research method requires an adequate quantity of reagents, which is determined experimentally. We determined the quantity of MLNCs required to detect HC protein material by SDS-PAGE. For this purpose, two, four, or eight identical preparations of MLNC/HCs were combined. After washing the unbound serum components and concentrating the MLNC/HC pellets, the presence of proteins was analyzed using Laemmli gel electrophoresis (Figure 8). When two MLNC preparations were simultaneously applied to a gel pocket, two bands were weakly detected (marked by the arrow in Figure 8A). Apparently, these are the main proteins that bind to the surface of MLNC.

When the number of preparations of MLNC/HCs increased to four or eight, the number of detected protein components increased. In addition to the previously identified “major” bands (marked with arrows, Figure 8B), many minor bands with different intensities of Coomassie staining appeared (marked with curly brackets, Figure 8B). Interestingly, in the control lane containing FBS, proteins corresponding to minor bands in lanes 1 and 2 (marked with curly brackets, Figure 8B) were not detected. This indicates that, exploiting the long time of protein desorption from the surface of MLNC, we “catch” proteins from the FBS solution that are present in the serum in lower concentrations than albumin and globulin. The reason for such a strong interaction between the hydrophilic charged surface of MLNC and some serum proteins is apparently hydrogen and electrostatic bonds [6]. 

The results obtained allow us to conclude that the proposed methods for isolating composite nanoparticles bearing a hard protein corona and washing them from unbound serum components are effective. Based on the hard protein corona isolation approach, we developed an approach to isolate the complete protein corona.

### 3.3. Obtaining MLNCs with Full Protein Corona

The composition of the SC is extremely variable due to the fact that its proteins, unlike the HC, bind to the surface of nanoparticles reversibly [6,17]. In this regard, the release of nanoparticles from biological fluids can lead to both partial and complete loss of SC proteins. Obviously, to study the composition and properties of a SC, it is necessary to anchor it on a HC tightly bound to the surface of the MLNC.

#### 3.3.1. Cross-Linker Design

**Figure 9:**
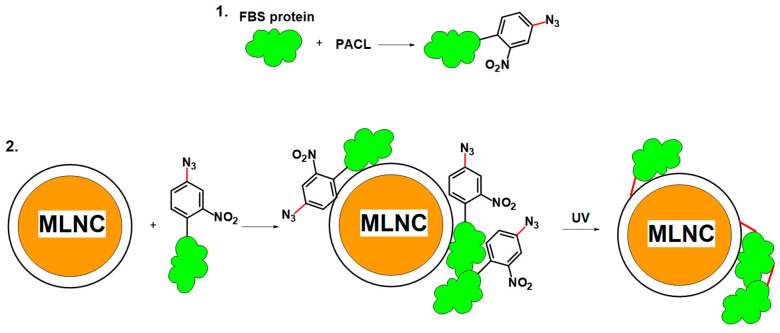
Scheme for obtaining MLNC with a fixed full corona. (**1**)—modification of free serum protein with PACL; (**2**)—adsorption of modified proteins to the MLNC surface and binding of modified proteins under UV irradiation with the MLNC surface.

We propose to use PACL to anchor SC proteins to the surface of MLNCs. PACL contains two functional groups; due to the first group, PACL binds to reactive groups on the surface of serum proteins; the second is photoactivated under the influence of UV radiation, which leads to cross-linking of both spatially close proteins on the MLNC surface and proteins with lipids on the MLNC surface. At the first stage, modification of serum proteins was carried out (Figure 9). Then, MLNCs were incubated with the modified serum, after which the resulting suspension was irradiated to fix the proteins on the surface of the nanoparticles (Figure 9). MLNCs with a fixed protein, corona, were purified using the method described above.

The following derivatives can be used as one reactive group to modify protein molecules: N-hydroxysuccinimide ester, imino ester, thiol group, and maleimide residue [18]. It is important that the conditions for introducing the modification and changes caused by the modification do not lead to structural changes in the modified proteins, since this can affect their sorption properties and, accordingly, the composition of SC. Therefore, the N-hydroxysuccinimide ester, imino ester, and thiol group were rejected. N-hydroxysuccinimide ester and imino ester were rejected because the modification conditions required a change in the acidity of the medium. This modification also leads to a decrease in the positive charge of proteins due to the modification of free amino groups. The use of PACLs containing a thiol group can also lead to changes in the structure of the modified protein due to the reduction of disulfide bridges. We settled on the maleimide group, which reacts with free thiol groups of cysteine under neutral pH values. As a rule, there are few free thiol residues on the surface of proteins. For example, bovine serum albumin (BSA) contains 35 cysteine residues, but only one residue is subject to modification since the rest form 17 intramolecular disulfide bridges [19].

The photoreactive group (the second PACL group) interacts with target molecules under UV exposure. An “ideal” photoreactive group should be highly reactive, able to bind to any type of residue, be stable in the dark, and have selectivity to a specific wavelength of light that does not cause photolytic damage to the biological sample. Moreover, the reaction between the photoreactive group and proteins should lead to stable products that can be further isolated. Arylazides, diazarines, and benzophenones are also used as photoreactive groups [20]. However, diazarins can react with target molecules in the dark, and the reaction with benzophenones is reversible, so the most common derivatives are arylazides [21]. We chose a nitroaryl azide derivative as the photoreactive group. 

**Figure 10:**
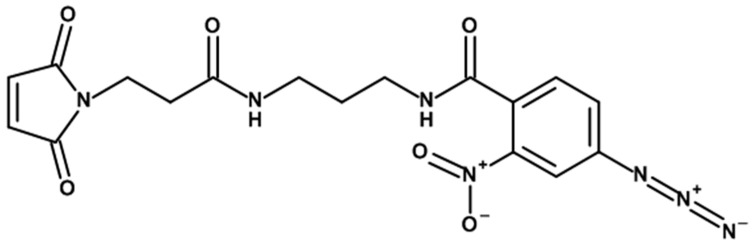
Structure of the PACL used in this work.

Next, the length of the linker was determined, which should connect the two reactive groups of PACL to each other. Since we have to work with a medium that is heterogeneous in protein composition, the length of the linker must ensure cross-linking of the modified protein on the MLNC surface with a nearby protein or other serum component, regardless of the topology of their surfaces. Excessive linker length may promote the binding of unsorbed proteins that are not part of the HC or SC, which may result in the accumulation of false-positive results. We used a short hydrophobic linker with a length of 22 Å; the structure of the PACL used in this work is presented in Figure 10.

#### 3.3.2. Modification of Serum Proteins

**Figure 11:**
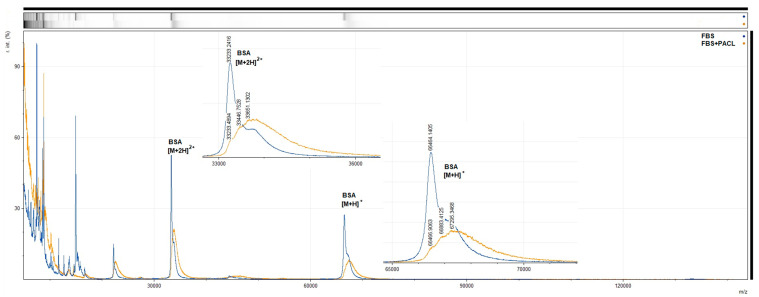
MALDI-TOF analysis of modified serum. The image shows two superposed spectra: the spectrum of FBS—blue curve; and the spectrum of PACL-modified FBS—orange curve. The figure also contains enlarged views of the peaks associated with BSA.

We determined the optimal conditions for modifying serum proteins using PACL: a 10-fold excess of PACL and a condensation time of two hours at room temperature. The effectiveness of the modification was assessed using mass spectrometry by measuring the change in the mass of serum albumin. Several overlapping peaks were observed in the mass spectrum (Figure 11). The first corresponds to the mass of BSA (66,466.9 Da), indicating its incomplete modification or the overlap of the peak of the doubly charged albumin dimer [22]. It should be noted that the contribution of this peak is insignificant. Some BSA molecules carry two modifications ((67,294.3 – 66,463.1)/414.4 ≈ 2), which indicates the occurrence of a side reaction at the amino groups of BSA. The mixture contains the mono-addition product PACL (66,882.4 Da); however, due to the side reactions occurring at the amino groups, it is impossible to say whether it is the product of the reaction of the cysteine or lysine residue of albumin with PACL.

We consider the modification conditions we have chosen to be effective. The presence of a peak corresponding to the initial mass of albumin itself is logically associated with the dimerization of the latter. Dimerization occurs due to the formation of a disulfide bridge between two free thiol cysteine residues in the albumin molecule. Dimerized albumin molecules should not undergo modification reactions. Consequently, under these conditions, exhaustive modification of free cysteines occurs. In addition, the presence of a non-specific reaction at lysine residues will also lead to the modification of non-thiol-containing proteins. 

Analysis of the modified serum did not show the presence of peaks associated with the formation of cross-links between proteins.

We assume that with the chosen length of the PACL linker and the efficiency of protein modification under established optimal conditions, cross-linking between the modified protein and the rest of the serum components will occur not in solution but when the proteins are brought together due to sorption on the MLNC surface.

**Figure 12:**
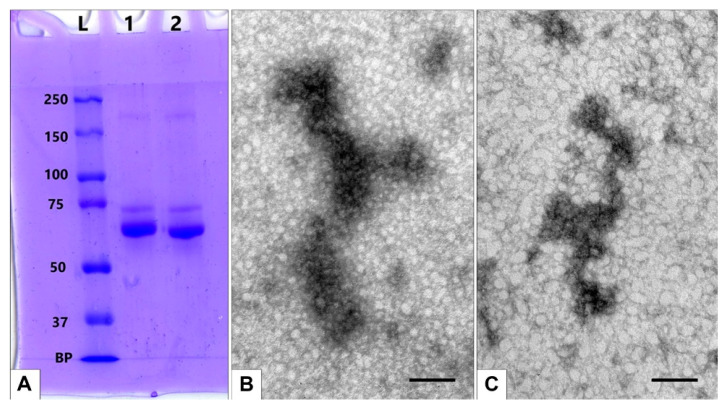
Absence of serum component aggregation without the presence of MLNCs. (**A**)—scanned SDS-PAGE image: L—protein molecular weight marker; 1—FBS modified by PACL and UV-irradiated; 2—FBS. BP—bromophenol blue. (**B**,**C**)—representative images of FBS proteins: (**B**)—solution of 10% FBS. (**C**)—solution of 10% FBS after treatment with PACL and irradiation. TEM, negative staining. The length of the scale bars corresponds to 50 nm.

Otherwise, we should observe the aggregation of serum components without the presence of MLNCs. To ensure the absence of such aggregation, we irradiated a 10% modified serum under the same conditions as the MLNC suspensions and analyzed it by gel electrophoresis and TEM. A slight decrease in the mobility of the band corresponding to albumin was observed on the SDS-PAGE gel, which is associated with intramolecular cross-linking. Additional bands with increased molecular weight did not appear during the irradiation of 10% FBS modified with PACL (Figure 12A). TEM analysis also did not reveal changes in the structure of the serum after photomodification and irradiation; shapeless, loose accumulations of electron-dense material were observed on negatively stained grids (Figure 12B,C). 

#### 3.3.3. Interaction of Modified Serum with MLNCs

**Figure 13:**
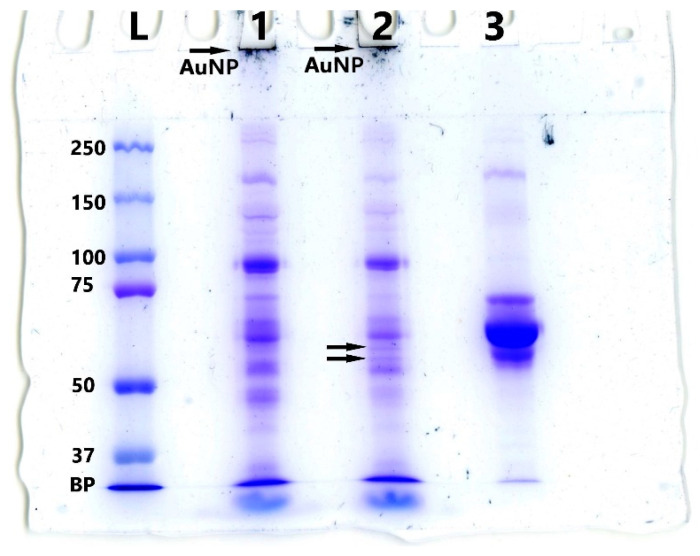
Scanned SDS-PAGE image: L—protein molecular weight marker; 1—MLNC + FBS isolated from GS; 2—MLNC + FBS/PACL isolated from GS; 3—FBS. BP—bromophenol blue.

Under the chosen conditions, the serum was modified with PACL and then incubated with the initial MLNC preparation. Incubation was followed by UV irradiation at a wavelength of 310 nm and an intensity of 8.2 mW/cm^2^ for 1 min, followed by separation in GS. The proteins of the corona of MLNCs were washed from unbound serum components and analyzed by SDS-PAGE. Incubation of MLNCs in PACL-modified serum followed by UV irradiation resulted in the appearance of additional bands, indicated by arrows on SDS-PAGE when analyzing the protein corona (Figure 13).

The appearance of new bands confirms the presence of PACL-modified proteins in the corona, as well as the formation of cross-links with proteins spatially close to them on the surface of MLNCs. Cross-link formation changes electrophoretic mobility.

#### 3.3.4. Detection of Full Corona Formation 

There is a possibility that protein aggregates may form in PACL-modified serum due to irradiation, and we conducted additional experiments to detect such aggregates.

**Figure 14:**
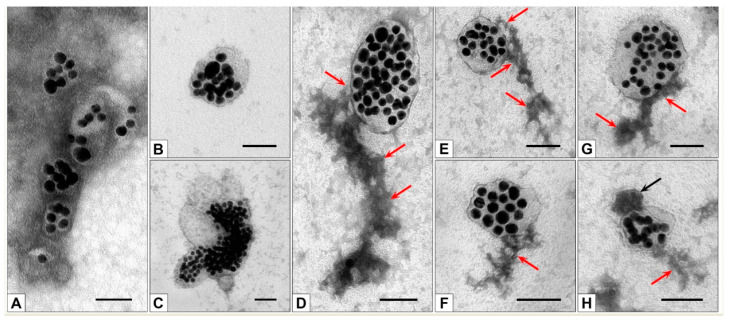
Representative images of MLNCs. (**A**)—MLNCs in a solution of 10% FBS. (**B**)—MLNC was incubated with unmodified serum and irradiated with UV. (**C**)— MLNC was incubated with FBS, modified with PACL, and irradiated with UV. (**D**–**H**)—MLNCs treated with modified serum and irradiated with UV. The corona associated with the surface of the MLNCs is shown by the red arrow. (**H**)—an electron-dense particle (black arrow) adsorbed on MLNC bearing a corona. TEM, negative staining. Length of scale bars corresponds to 50 nm.

(1) We incubated MLNCs with unmodified 10% FBS and irradiated them with UV. The TEM study revealed only “naked” particles; no signs of a corona on their surface were detected (Figure 14B). The isolated particles did not differ from similar ones bearing a hard corona (Figure 7C,D), which means that HC on MLNCs is not seen in TEM on negatively stained samples. 

(2) We incubated MLNCs with modified and irradiated 10% FBS. Again, we observed “naked” particles without signs of a corona on the surface (Figure 14C). It should be noted that MLNCs in this preparation looked larger. 

TEM examination of MLNCs treated with PACL-modified serum and irradiated with UV revealed distinct changes in their structure, apparently associated with soft corona formation (Figure 14D–H). 

Coronas of different sizes appear on the surface of MLNCs incubated with modified serum and irradiated with UV, usually in the form of a “tail,” often attached to the surface of MLNCs by a narrow “bridge” (Figure 14D–H). The substance of the “tails” looked structured; depending on the thickness of the “tail,” “outgrowths” and “twigs” were observed. The “tails” have clear boundaries and a heterogeneous structure; the electron density of their substance varies from medium to high. This observation reflects the presence of different proteins in the corona composition. 

We are the first to obtain images of the corona fixed to the surface of NPs using photomodification. Similar studies have not been published, and we have nothing to compare the structure of the corona with. The corona formed on MLNCs is not similar to the coronas in the form of a uniform cloud around metal and polymer NPs presented in a number of publications [10,23,24,25]. We believe that the shape of the corona on the MLNC is due to the uneven surface properties of the lipid-peptide envelope of this nanoconstruct, as well as the high diversity of proteins in the serum. 

Evidence in favor of this assumption comes from recent publications on corona studies on extracellular vesicles (EVs), which are naturally occurring lipid-enveloped NPs and play important roles in many physiological processes. The uneven distribution of transmembrane proteins in the EV membrane, shown by super-resolution fluorescence microscopy, may determine the uneven binding of environmental proteins to the membrane, followed by “uneven” corona on the surface of the vesicles described [26]. 

The formation of a corona consisting of large protein aggregates randomly sorbed on the surface of EVs was demonstrated using confocal microscopy [27]. The belonging of these aggregates to the protein corona was confirmed by immunoelectron microscopy and changes in membrane structure of naked and coronated EVs on ultrathin sections. These results reflect the complexity of the interaction of the lipid membranes of natural EVs with environmental proteins and are not surprising. We think that the structure of the corona observed on MLNC is consistent with the corona variants described in [27]. 

On the surface of MLNCs bearing a corona, spherical electron-dense particles (20–50 nm) are sometimes found (Figure 14H). In the center of the particles, a “light” core, surrounded by an uneven layer of high electron density, can be recognized. The structure and size of these particles make it possible to identify them as lipoproteins (non-vesicles) present in serum and other biological fluids [28,29].

The appearance of a full corona on the surface of the MLNCs after UV irradiation allows us to conclude that it was the fixation of the components of the soft corona to the hard (“invisible” in TEM) that made it possible to obtain a visible, complete corona.

The results of the TEM study fully correspond to the data from DLS studies (Table 2). 

The DLS study showed that contact of MLNCs with FBS, isolation, and washing (Table 2, lines 1, 2) resulted in an increase in hydrodynamic diameter (HD) from 210.4 ± 86.79 nm to 249.9 ± 124.2 nm and a change in the ζ-potential value from −35.3 ± 5.6 mV to −43.0 ± 5.2 mV. Low values of the polydispersity index (PDI) were maintained (less than 0.2). The observed changes in the HD and ζ-potential indicate changes in the surface layer of nanoparticles caused by the formation of HC, which is not visualized in TEM.

Irradiation of a suspension of MLNCs in unmodified serum with UV resulted in smaller values of HD (225 ± 97.83 nm) and ζ-potential (−38.5 ± 5.2 mV) (Table 2, line 3). This may be due to changes in the structural and dimensional characteristics of proteins under the influence of UV irradiation [30]. A corona is not visualized in TEM in this sample (Figure 14B).

Unexpectedly high HD was recorded for MLNCs isolated from GS after incubation with UV-irradiated PACL-modified FBS (Table 2, line 4). The observed increase in the HD of MLNCs to 288.4 ± 116.8 nm is due to some of their changes with the required additional concentration of the initial preparation. Enlargement of MLNCs sizes in this experiment was also noted by TEM. However, an increase in the HD of these MLNCs (Table 2, line 4) did not lead to a change in ζ-potential (−37.2 ± 5.1 mV), which corresponds to the absence of SC on their surface. At the same time, irradiation of MLNCs and modified serum leads to a decrease in ζ-potential (Table 2, line 5), which indicates the formation of SC detected in TEM (Figure 14D–H).

Thus, we detected a full corona, composed of HC and SC, on the surface of MLNCs incubated with PACL-modified FBS and UV-irradiated. The full corona is the result of photofixation of spatially close proteins on the lipid surface of MLNCs, not the sorption of spontaneously formed protein aggregates.

## 4. Discussion

We are the first to demonstrate the feasibility of fixing a full-protein corona on the lipid surface of composite nanoparticles (MLNCs) using PACL. Previously, a method of soft corona fixation using click chemistry was proposed [10], which requires separate modification of hard corona and serum proteins. Our approach involves PACL modification of only serum proteins, which are then sorbed onto the surface of MLNCs, form hard and soft coronas, and are fixed to the surface under UV irradiation. Mass spectrometric analysis showed that the serum components carried one or two modifications. After incubation of MLNCs in modified serum and UV irradiation under chosen conditions, particles bearing a full corona were isolated. MLNCs were visualized using TEM at each stage of this study. The formation of a HC did not change the structure of MLNC/HCs compared to MLNCs, while the full corona was clearly visualized. A full corona under the selected conditions and with a photomodifier does not completely cover the surface of the particles but forms aggregates with linear dimensions comparable to the particle sizes. Apparently, such sorption of corona proteins is due to the heterogeneity of the lipid-peptide membrane, which may be caused by clustering of its components. 

## 5. Conclusions

For the first time, the use of a photoactivatable cross-linker made it possible to capture and visualize the full protein corona on the surface of a nanocomposite enveloped with a lipid layer. The proposed version of the full corona fixation technique can be applied to various types of particles as well as types of biological fluids.

## Figures and Tables

**Table 1:** Efficiency of washing MLNCs from free proteins.

Number of Washes	RFU(Cy5)/RFU(albumin+Cy5-albumin) × 100%
Supernatant after MLNC isolation	0.686 ± 0.059
1st wash	0.021 ± 0.012
2nd wash	0.015 ± 0.014
3rd wash	0.002 ± 0.001
4th wash	not detected

**Table 2:** Hydrodynamic diameter and ζ-potential of MLNCs isolated from GS.

Line	Sample	Size ± SD (d.nm)	ζ-Potential ± SD (mV)	PDI
1	MLNC	210.4 ± 86.79	−35.3 ± 5.6	0.148
2	MLNC + FBS	249.9 ± 124.2	−43.0 ± 5.2	0.176
3	MLNC + FBS UV-irradiated	225 ± 97.83	−38.5 ± 5.2	0.193
4	MLNC&FBS/PACL/UV	288.4 ± 116,8	−37.2 ± 5.1	0.193
5	MLNC + FBS/PACL	242.3 ± 120.0	−41.2 ± 5.8	0.175

## Data Availability

Data are available on request from the corresponding authors.

## References

[1] García-Álvarez R., Vallet-Regí M. (2021). Hard and Soft Protein Corona of Nanomaterials: Analysis and Relevance. Nanomaterials.

[2] Kruszewska J., Zajda J., Matczuk M. (2021). How to effectively prepare a sample for bottom-up proteomic analysis of nanoparticle protein corona? A critical review. Talanta.

[3] Gunawan C., Lim M., Marquis C.P., Amal R. (2014). Nanoparticle–protein corona complexes govern the biological fates and functions of nanoparticles. J. Mater. Chem. B.

[4] Ren J., Andrikopoulos N., Velonia K., Tang H., Cai R., Ding F., Ke P.C., Chen C. (2022). Chemical and Biophysical Signatures of the Protein Corona in Nanomedicine. J. Am. Chem. Soc..

[5] Xiao Q., Zoulikha M., Qiu M., Teng C., Lin C., Li X., Sallam M.A., Xu Q., He W. (2022). The effects of protein corona on in vivo fate of nanocarriers. Adv. Drug Deliv. Rev..

[6] Nienhaus K., Nienhaus G.U. (2023). Mechanistic Understanding of Protein Corona Formation around Nanoparticles: Old Puzzles and New Insights. Small.

[7] Blume J.E., Manning W.C., Troiano G., Hornburg D., Figa M., Hesterberg L., Platt T.L., Zhao X., Cuaresma R.A., Everley P.A. (2020). Rapid, deep and precise profiling of the plasma proteome with multi-nanoparticle protein corona. Nat. Commun..

[8] Chu Y., Tang W., Zhang Z., Li C., Qian J., Wei X., Ying T., Lu W., Zhan C. (2021). Deciphering Protein Corona by scFv-Based Affinity Chromatography. Nano Lett..

[9] Weber C., Simon J., Mailänder V., Morsbach S., Landfester K. (2018). Preservation of the soft protein corona in distinct flow allows identification of weakly bound proteins. Acta Biomater..

[10] Mohammad-Beigi H., Hayashi Y., Zeuthen C.M., Eskandari H., Scavenius C., Juul-Madsen K., Vorup-Jensen T., Enghild J.J., Sutherland D.S. (2020). Mapping and identification of soft corona proteins at nanoparticles and their impact on cellular association. Nat. Commun..

[11] Epanchintseva A.V., Poletaeva J.E., Dovydenko I.S., Chelobanov B.P., Pyshnyi D.V., Ryabchikova E.I., Pyshnaya I.A. (2021). A Lipid-Coated Nanoconstruct Composed of Gold Nanoparticles Noncovalently Coated with Small Interfering RNA: Preparation, Purification and Characterization. Nanomaterials.

[12] Epanchintseva A.V., Poletaeva J.E., Dome A.S., Dovydenko I.S., Pyshnaya I.A., Ryabchikova E.I. (2022). Chemical Modifications Influence the Number of siRNA Molecules Adsorbed on Gold Nanoparticles and the Efficiency of Downregulation of a Target Protein. Nanomaterials.

[13] Shashkova V.V., Epanchintseva A.V., Vorobjev P.E., Razum K.V., Ryabchikova E.I., Pyshnyi D.V., Pyshnaya I.A. (2017). Multilayer associates based on oligonucleotides and gold nanoparticles. Russ. J. Bioorg. Chem..

[14] Poulsen K.M., Payne C.K. (2022). Concentration and composition of the protein corona as a function of incubation time and serum concentration: An automated approach to the protein corona. Anal. Bioanal. Chem..

[15] Milani S., Baldelli Bombelli F., Pitek A.S., Dawson K.A., Rädler J. (2012). Reversible *versus* Irreversible Binding of Transferrin to Polystyrene Nanoparticles: Soft and Hard Corona. ACS Nano.

[16] Winzen S., Schoettler S., Baier G., Rosenauer C., Mailaender V., Landfester K., Mohr K. (2015). Complementary analysis of the hard and soft protein corona: Sample preparation critically effects corona composition. Nanoscale.

[17] Mahmoudi M., Landry M.P., Moore A., Coreas R. (2023). The protein corona from nanomedicine to environmental science. Nat. Rev. Mater..

[18] Sinz A. (2006). Chemical cross-linking and mass spectrometry to map three-dimensional protein structures and protein–protein interactions. Mass Spectrom. Rev..

[19] Smith M.E.B., Caspersen M.B., Robinson E., Morais M., Maruani A., Nunes J.P.M., Nicholls K., Saxton M.J., Caddick S., Baker J.R. (2015). A platform for efficient, thiol-stable conjugation to albumin’s native single accessible cysteine. Org. Biomol. Chem..

[20] Murale D.P., Hong S.C., Haque M., Lee J.-S. (2017). Photo-affinity labeling (PAL) in chemical proteomics: A handy tool to investigate protein-protein interactions (PPIs). Proteome Sci..

[21] Preston G.W., Wilson A.J. (2013). Photo-induced covalent cross-linking for the analysis of biomolecular interactions. Chem. Soc. Rev..

[22] Naldi M., Baldassarre M., Nati M., Laggetta M., Giannone F.A., Domenicali M., Bernardi M., Caraceni P., Bertucci C. (2015). Mass spectrometric characterization of human serum albumin dimer: A new potential biomarker in chronic liver diseases. J. Pharm. Biomed. Anal..

[23] Izak-Nau E., Voetz M., Eiden S., Dusch A., Puntes V. (2013). Altered characteristics of silica nanoparticles in bovine and human serum: The importance of nanomaterial characterization prior to its toxicological evaluation. Part. Fibre Toxicol..

[24] Del Chantada-Vázquez M.P., López A.C., García-Vence M., Acea-Nebril A., Bravo S., Núñez C. (2020). Protein Corona Gold Nanoparticles Fingerprinting Reveals a Profile of Blood Coagulation Proteins in the Serum of HER2- Overexpressing Breast Cancer Patients. Int. J. Mol. Sci..

[25] Stewart M., Mulenos M.R., Steele L.R., Sayes C.M. (2018). Differences among Unique Nanoparticle Protein Corona Constructs: A Case Study Using Data Analytics and Multi-Variant Visualization to Describe Physicochemical Characteristics. Appl. Sci..

[26] Wolf M., Poupardin R.W., Ebner-Peking P., Andrade A.C., Blochl C., Obermayer A., Gomes F.G., Vari B., Maeding N., Eminger E. (2022). A functional corona around extracellular vesicles enhances angiogenesis, skin regeneration and immunomodulation. J. Extracell. Vesicl..

[27] Tóth E.Á., Turiák L., Visnovitz T., Cserép C., Mázló A., Sódar B.W., Försönits A.I., Petővári G., Sebestyén A., Komlósi Z. (2021). Formation of a protein corona on the surface of extracellular vesicles in blood plasma. J. Extracell. Vesic..

[28] Grigor’eva A.E., Dyrkheeva N.S., Bryzgunova O.E., Tamkovich S.N., Chelobanov B.P., Ryabchikova E.I. (2017). Contamination of exosome preparations, isolated from biological fluids. Biomed. Khim..

[29] Théry C., Witwer K.W., Aikawa E., Alcaraz M.J., Anderson J.D., Andriantsitohaina R., Antoniou A., Arab T., Archer F., Atkin-Smith G.K. (2018). Minimal information for studies of extracellular vesicles 2018 (MISEV2018): A position statement of the International Society for Extracellular Vesicles and update of the MISEV2014 guidelines. J. Extracell. Vesicles.

[30] Kristo E., Hazizaj A., Corredig M. (2012). Structural Changes Imposed on Whey Proteins by UV Irradiation in a Continuous UV Light Reactor. J. Agric. Food Chem..

